# Carbohydrate Stress Affecting Fruitlet Abscission and Expression of Genes Related to Auxin Signal Transduction Pathway in Litchi

**DOI:** 10.3390/ijms131216084

**Published:** 2012-11-29

**Authors:** Jian-Fei Kuang, Jian-Yang Wu, Hai-Ying Zhong, Cai-Qin Li, Jian-Ye Chen, Wang-Jin Lu, Jian-Guo Li

**Affiliations:** 1 State Key Laboratory for Conservation and Utilization of Subtropical Agro-Bioresources/Guangdong Key Laboratory for Postharvest Science, College of Horticulture, South China Agricultural University, Guangzhou 510642, China; E-Mails: jfkuang@scau.edu.cn (J.-F.K.); zhyxfy@126.com (H.-Y.Z.); chenjianye@scau.edu.cn (J.-Y.C.); wjlu@scau.edu.cn (W.-J.L.); 2 China Litchi Research Center, South China Agricultural University, Guangzhou 510642, China; E-Mails: wjiany065@163.com (J.-Y.W.); lcq_chin@163.com (C.-Q.L.); 3 College of Basic Education, Zhanjiang Normal University, Zhanjiang 524037, China

**Keywords:** *Litchi chinensis* Sonn., auxin responsive genes, auxin response factor, clone, gene expression, carbohydrate stress, fruitlet abscission

## Abstract

Auxin, a vital plant hormone, regulates a variety of physiological and developmental processes. It is involved in fruit abscission through transcriptional regulation of many auxin-related genes, including early auxin responsive genes (*i.e.*, auxin/indole-3-acetic acid (*AUX/IAA*), Gretchen Hagen3 (*GH3*) and small auxin upregulated (*SAUR*)) and auxin response factors (*ARF*), which have been well characterized in many plants. In this study, totally five auxin-related genes, including one *AUX/IAA* (*LcAUX/IAA1*), one *GH3* (*LcGH3.1*), one *SAUR* (*LcSAUR1*) and two *ARFs* (*LcARF1* and *LcARF2*), were isolated and characterized from litchi fruit. *LcAUX/IAA1*, *LcGH3.1*, *LcSAUR1*, *LcARF1* and *LcARF2* contain open reading frames (ORFs) encoding polypeptides of 203, 613, 142, 792 and 832 amino acids, respectively, with their corresponding molecular weights of 22.67, 69.20, 11.40, 88.20 and 93.16 kDa. Expression of these genes was investigated under the treatment of girdling plus defoliation which aggravated litchi fruitlet abscission due to the blockage of carbohydrates transport and the reduction of endogenous IAA content. Results showed that transcript levels of *LcAUX/IAA1*, *LcGH3.1* and *LcSAUR1* mRNAs were increased after the treatment in abscission zone (AZ) and other tissues, in contrast to the decreasing accumulation of *LcARF1* mRNA, suggesting that *LcAUX/IAA1*, *LcSAUR1* and *LcARF1* may play more important roles in abscission. Our results provide new insight into the process of fruitlet abscission induced by carbohydrate stress and broaden our understanding of the auxin signal transduction pathway in this process at the molecular level.

## 1. Introduction

Fruit abscission, occurring in advance of harvest, is characterized by the activation of a specific abscission zone (AZ) located between the pedicel and fruitlet, resulting in severe economic losses in many fruits [1,2]. The abscission is a highly regulated developmental process that is both influenced and activated by either internal cues or environmental conditions. Endogenous plant hormones have been implicated to contribute to the abscission of immature fruit, among which auxin is suggested to be a key regulator in fruit retention [3]. Spraying of auxins or synthetic auxins (e.g., 1-naphthaleneacetic acid (NAA) or 2,4-dichlorophenoxyacetic acid (2,4-D)) can efficiently reduce the fruit abscission by maintaining the cells adherence in the AZ and repress the activity of hydrolytic enzymes, such as cellulases which, in turn, promote fruitlet abscission by loosening the cell walls [4,5].

Auxin plays a pivotal role in the process of plant organ abscission. It is increasingly interesting to investigate the role of genes related to the auxin signal transduction pathway in this process. It is well documented that auxin stimulates the rapid transcription of a set of genes called primary or early auxin responsive genes including *Aux/IAA*, *GH3*, *SAUR*[6–8]. The AUX/IAA proteins are short-lived nuclear proteins that function as repressors of auxin-induced transcription, and auxin promotes the degradation of this large family of transcriptional regulators, leading to diverse downstream responses [9,10]. *GH3* genes encode IAA-conjugating enzymes, which could act as feedback regulators by reducing free auxin levels [11], while *SAUR* genes code for highly conserved short-lived small proteins and likely function in cell expansion, hypocotyls and stamen filament elongation [12,13]. The promoter regions of these primary/early auxin-responsive genes contain one or more auxin-responsive elements (AuxRE) conferring auxin responsiveness. The identification of the AuxRE sequence led to the isolation of the *Arabidopsis Auxin Response Factor1* (*ARF1*) gene which was shown to bind with specificity to AuxRE [14,15]. *ARF* transcription factors regulate many responses to auxin, including embryo patterning, vascular formation, phototropism and gravitropism [16,17]. Recently, it was found that expression of *Ce-IAA* in the AZ was negatively correlated with floret bud abscission in *Cestrum* cut flower [18]. Similar correlation between the effectiveness of auxin in delaying abscission and induction of *Aux/IAA* gene expression were also found in *Cestrum elegans*[19] and *Mirabilis jalapa*[20]. In tomato, *SlIAA* genes play important roles in delaying floral pedicel abscission, with *SlIAA1*, *9* and *12* required for the completion of ethylene-induced abscission and *SlIAA4*, *6*, *7*, *8*, *14*, *16*, *17* and *29* important for calcium-delayed abscission [21]. Tomato *GH3* effectively represses the delayed abscission induced by IAA, while *SAUR* may serve as a marker of IAA level throughout the abscission process [21]. In Arabidopsis, two *ARF* genes, *ARF1* and *ARF2*, along with other *ARF* genes, *Nonphototropic Hypocotyl4 (NPH4)/ARF7* and *ARF19*, might act together to regulate senescence and floral organ abscission [22].

The substantial production of Litchi (*Litchi chinensis* Sonn.), an important economic fruit crop widely grown in southern China and other southeast Asian areas [23], has been challenged by excessive fruit drop, one of the major factors causing irregular cropping or alternate bearing. There are two or three distinct abscission periods depending on cultivar during each reproductive cycle, typically a year [24]. The first period of abscission occurs at the end of full female bloom and lasts for about a month; the second abscission wave starts one or two weeks later and lasts for about two weeks [25]. The third drop wave, lasting for about one week, takes place two to three weeks before fruit maturation [24]. Generally, only 2% to 5% of the initial female flowers develop into mature fruits [25]. The peak and periodicity of fruit drop are affected by environmental and/or internal factors, for instance, spells of heavy overcast and rainy weather. Low temperature or drought during the flowering and fruit-setting period always induces sever fruit abscission. Yuan and Huang (1988) demonstrated that litchi fruit-set relied greatly upon current photosynthates and the fruit abscission multiwaves were in parallel with the upsurges of abscissic acid (ABA) in the seed, while IAA seemed to be antagonistic [26]. Basipetal IAA flux through AZ prevents abscission by rendering the AZ insensitive to ethylene [20,27]. Recently, microarray analysis revealed that expressions of abscission-related genes were initiated by flower removal and leading to pedicel abscission were inhibited by IAA application, confirming that ethylene sensitivity in the AZ is associated with altered expression of auxin-regulated genes resulting from auxin depletion [28]. In addition, auxin can also influence carbohydrate availability to affect fruit abscission. For example, application of synthetic auxin 3,5,6-TPA (3,5,6-trichloro-2-pyridil-oxyacetic acid) stimulated carbohydrate accumulation in developing fruitlets to reduce the dropping of citrus fruit [29]. Synthetic auxins including NAA, 2,4-D, 2,4,5-T (2,4,5-trichlorophenoxyacetic acid) and 2,4,5-TP (2-(2,4,5-trichlorophenoxy) propionic acid), were also widely used to prevent fruit abscission in litchi management practices [30,31]. As auxin response is known to be mediated in part by altering gene expression, characterization of genes related to the auxin signal transduction pathway is of particular significance for understanding the molecular basis of auxin action [18,20,22].

Therefore, in this study, genes related to auxin signaling were firstly isolated from litchi fruit, and their expression patterns in response to girdling plus defoliation treatment—an approach to completely block the carbohydrate transport to fruitlets and induce severe carbohydrate stress for fruit development [32,33]—were investigated. Our results expand our understanding of carbohydrate stress-induced fruitlet abscission at the molecular level and have theoretical implications for exploring effective strategies to regulate litchi fruit abscission.

## 2. Results

### 2.1. Isolation of *AUX/IAA*, *GH3*, *SAUR* and *ARF* Genes from Litchi Fruit

**Figure 1:**
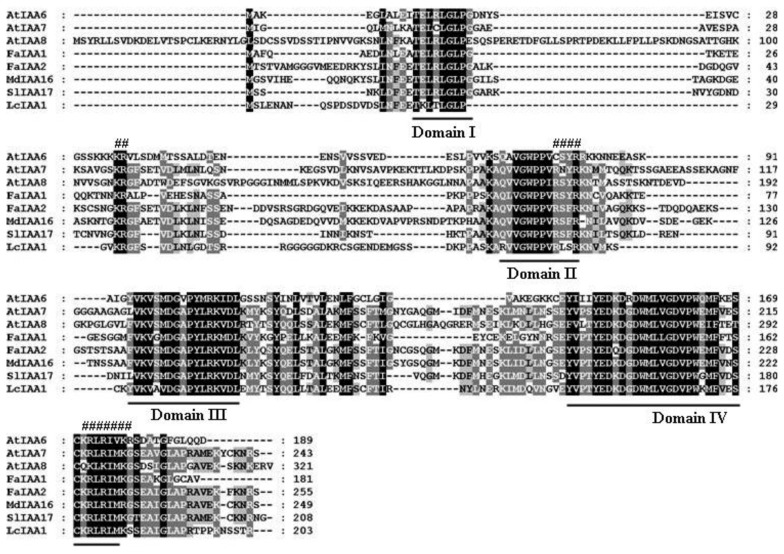
Alignment of the deduced amino acid sequence of litchi LcAUX/IAA1 and its respective homologs from *Arabidopsis* (AtIAA6, Q38824; AtIAA7, Q38825; AtIAA8, Q38826), strawberry [34], and tomato (SlIAA17, AEX00360). The shading indicates sequence similarities of 100% (black), or 80% (gray) among AUX/IAA proteins. Multiple alignments was done by Clustal W and viewed with Boxshade program. The four conserved domains in *Aux/IAA* proteins were underlined. A bipartite nuclear localization signal (NLS) between domains I and II, and a MAT α2-like NLS present at the end of domain IV were marked with “#”.

**Figure 2:**
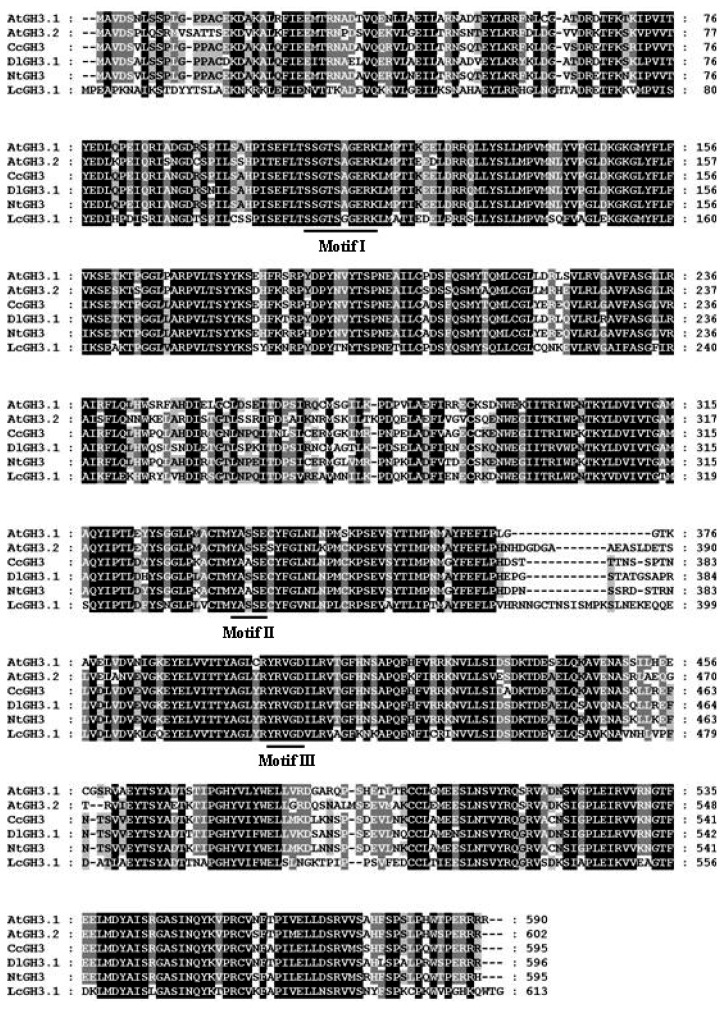
Alignment of the deduced amino acid sequence of litchi LcGH3.1 and its respective homologs from *Arabidopsis* (AtGH3.1, NM_127059; AtGH3.2, NM_119902), pepper (CcGH3, AY525089), longan (DlGH3.1, ADK27716) and tobacco (NtGH3, AF123503). The shading indicates sequence similarities of 100% (black), or 80% (gray) among GH3 proteins. Multiple alignments was done by Clustal W and viewed with Boxshade program. The three functional motifs (I, II and III) involved in ATP/AMP binding were underlined.

**Figure 3:**
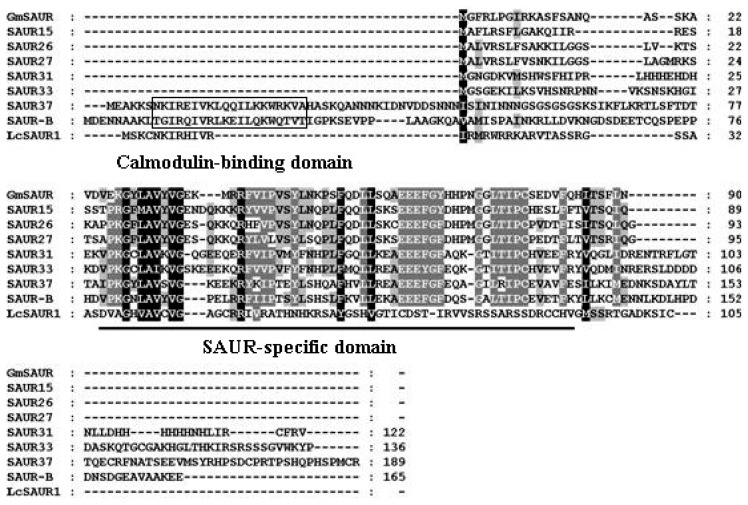
Alignment of the deduced amino acid sequence of litchi LcSAUR1 and its respective homologs from *Arabidopsis* (SAUR15, NP_195596; SAUR26, NP_187035; SAUR27, NP_187034; SAUR31, NP_567196; SAUR33, NP_191749; SAUR37, NP_194860; SAUR-B, NP_197581), and *Glycine max* (GmSAUR, S44175). The shading indicates sequence similarities of 100% (black), or 80% (gray) among SAUR proteins. Multiple alignments was done by Clustal W and viewed with Boxshade program. The SAUR specific domain is underlined. The calmodulin-binding domain is indicated by boxed.

**Figure 4:**
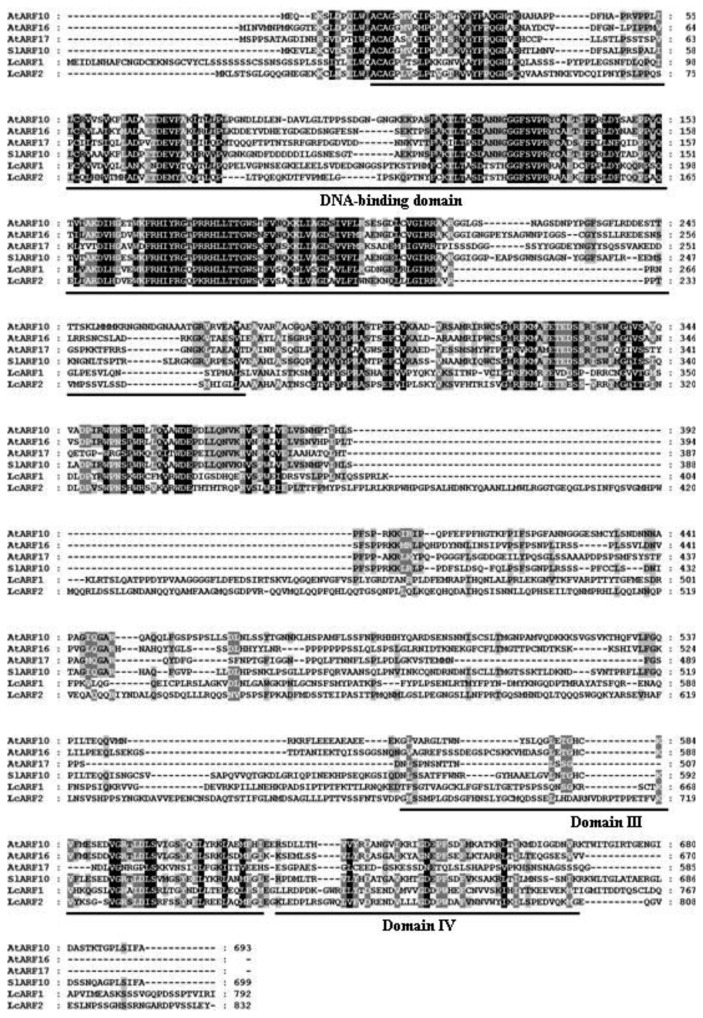
Alignment of the deduced amino acid sequence of two litchi ARFs (LcARF1 and LcARF2) and their homologs from *Arabidopsis* (AtARF10, NP_180402; AtARF16, NP_567841; AtARF17, NP_565161), and tomato (SlARF10, NP_001234796). The shading indicates sequence similarities of 100% (black), or 80% (gray) among ARF proteins. Multiple alignments was done by Clustal W and viewed with Boxshade program. DNA-binding domain and protein–protein interaction domains Box III and Box IV are underlined.

Full-Length sequences of one *AUX/IAA* (*LcAUX/IAA1*), one *GH3* (*LcGH3.1*), one *SAUR* (*LcSAUR1*) and two *ARF* (*LcARF1* and *LcARF2*) genes were isolated and characterized from litchi fruit for the first time. Sequence analysis of the full-length *LcAUX/IAA1* cDNA revealed that it bore a 609 bp open reading frame (ORF) encoding a predicted polypeptide of 203 amino acids, with the predicted molecular weights of 22.67 kDa and pI of 8.27. Multiple sequence alignment showed that LcAUX/IAA1 contained four conserved domains (I, II, III and IV), which are typical motifs for the Aux/IAA proteins (Figure 1) [7]. In addition, the basic amino acids located in between the domains I and II (.KR.RSYR..) constitute a bipartite nuclear localization signal (NLS) and a basic cluster KRLRIMK, resembling Simian Virus 40 (SV40) and a Mat a2-like NLS, which is present at the end of domain IV (Figure 1) [34]. The litchi *GH3* gene contains an ORF of 613 amino acids, with the predicted molecular weights of 69.20 kDa and pI of 6.81. The deduced protein contains three short conserved sequence motifs, GTSAGERK (Motif 1), YASSE (Motif 2) and YRVGD (Motif 3), which are involved in Adenosine Triphosphate (ATP)/Adenosine Monophosphate (AMP) binding as characteristic of the acyladenylate/thioester-forming enzyme super family (Figure 2) [35–37]. The *LcSAUR1* is 426 bp in length, which is deduced to encode a polypeptide of 142 amino acids with a predicted molecular weight of 11.40 kDa and pI of 11.52. Figure 3 shows that the LcSAUR1 protein contains a conserved SAUR specific domain (SSD) of approximately 60 residues in the central region like other reported SAUR proteins [38], but it does not contain the calmodulin-binding domain (CBD) that is present within the *N*-terminal extensions of SAUR-B and SAUR37 [38,39]. As for the two ARFs, their deduced proteins of *LcARF1* and *LcARF2* are of different lengths (792 and 832 aa), molecular masses (88.20 and 93.16 kDa), and isoelectric points (6.47 and 6.03). LcARF1 and LcARF2 show the typical features of auxin response factors, such as one *N*-terminal DNA-binding domain and two *C*-terminal domains (“Box III” and “Box IV”), which are implicated in protein dimerization (Figure 4). Interestingly, the middle regions of the two litchi ARFs (LcARF1 and LcARF2) are rich in proline, serine and threonine residues, a feature shared by AtARF1, which can repress auxin-induced, AuxRE-mediated gene expression, suggesting that they might function as transcription repressors [40].

### 2.2. Effect of Girdling Plus Defoliation on the Abscission of Litchi Fruitlet and Endogenous IAA Content

**Figure 5:**
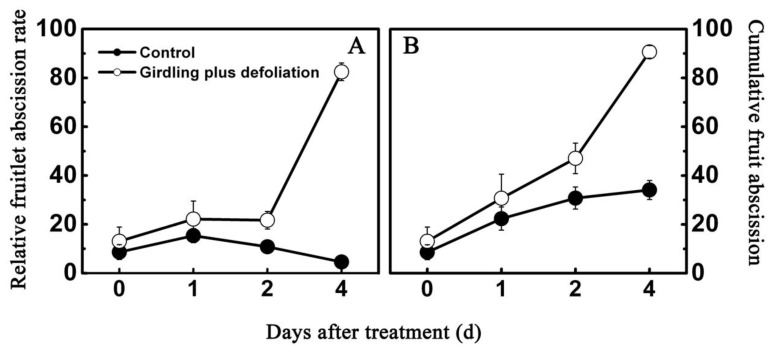
Effect of girdling plus defoliation treatment on fruitlet abscission of litchi. (**A**) Change of relative fruit abscission rate within four days after the girdling plus defoliation treatment; (**B**) Change of cumulative fruit abscission rate within four days after the girdling plus defoliation treatment. Each value represented the means of three biological replicates from three different trees, with the standard error (SE) indicated by vertical bars.

**Figure 6:**
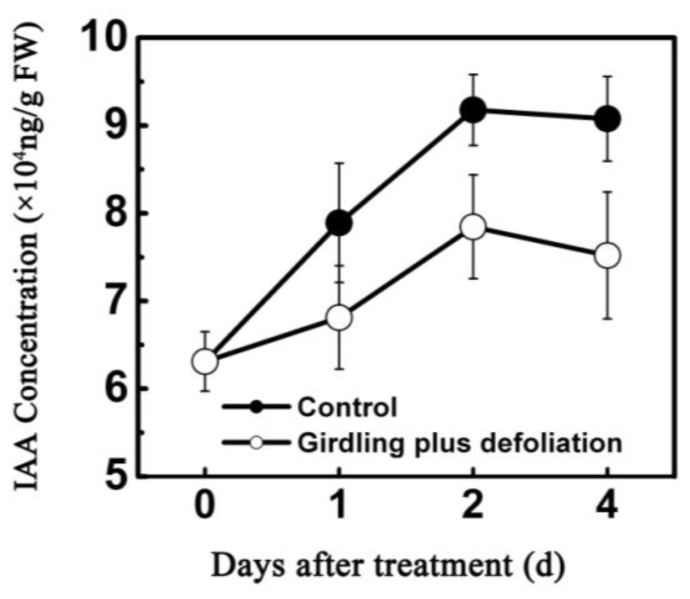
Quantitative assay of free IAA content in fruitlet of control and girdling plus defoliation treatment. Samples from 0, 1, 2 and 4 days after the treatment were used for Enzyme-linked immunosorbent assay (ELISA) analysis. Each value represented the means of three biological replicates from three different trees, with the standard error (SE) indicated by vertical bars.

As shown in Figure 5A, relative fruitlet abscission rate for the control remained nearly unchanged from 1 to 4 days, with a small peak at 1 days followed by slight decrease thereafter, whereas the treatment of girdling combined with defoliation led to a significant increase in the rate of fruit abscission during the same period, especially at 4 days when a striking increase was observed. Similar pattern of abscission was observed in total fruit drop (Figure 5B). The treatment induced a higher level of fruitlet drop, with two-fold fruitlet abscised when compared to the control at 4 days. However, endogenous IAA content less accumulated in the treated fruitlet, with 17.2% lower than that of the control at 4 days after treatment (Figure 6), suggesting that girdling plus defoliation treatment accelerates the fruit drop probably *via* reducing auxin availability in fruitlet.

### 2.3. Effect of Girdling Plus Defoliation on the Expression of Litchi *AUX/IAA*, *GH3*, *SAUR* and *ARF* Genes

**Figure 7:**
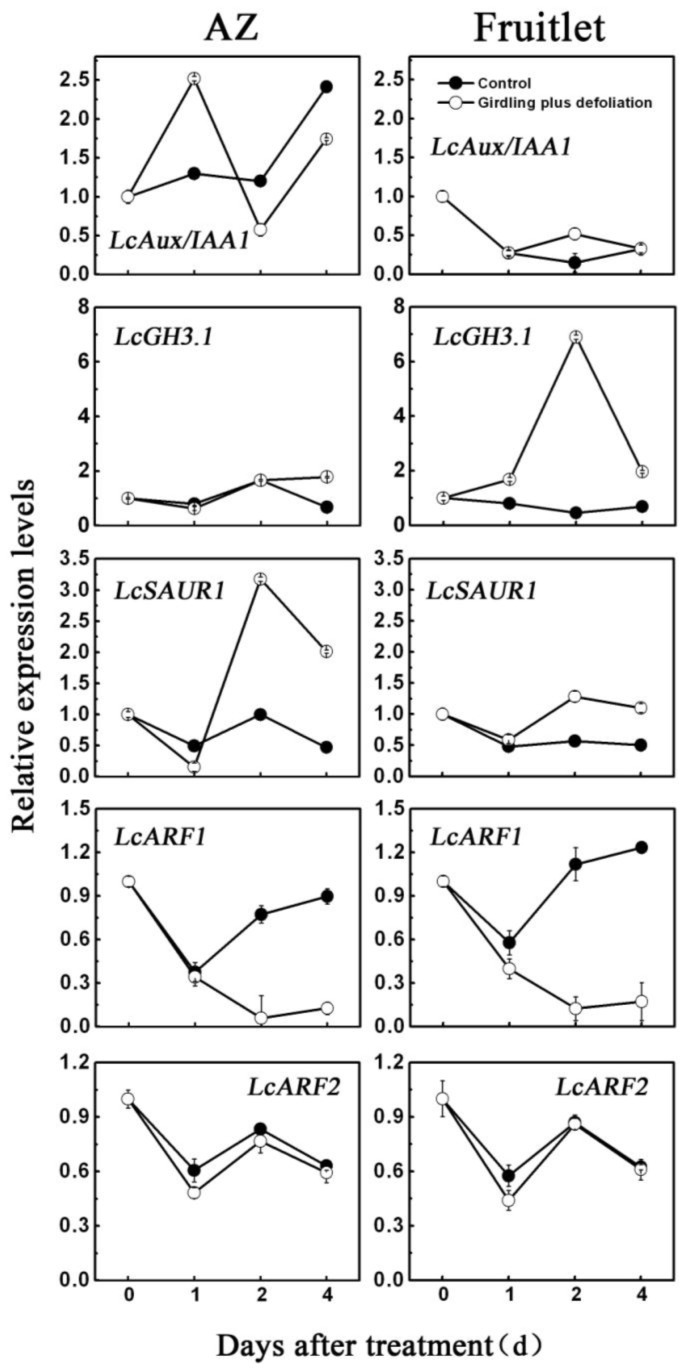
Expression analysis of *LcAUX/IAA1*, *LcGH3.1*, *LcSAUR1*, *LcARF1* and *LcARF2* mRNAs in the fruitlet abscission zone (AZ) and fruitlet of litchi after the treatment of girdling plus defoliation. Total RNA were isolated from fruit AZ and fruitlet collected at 0, 1, 2, and 4 days after the treatment, and subjected to qRT-PCR analysis using *Litchi chinensis actin (LcACTIN)* as an internal control. Expression levels of each gene are expressed as a ratio relative to the treatment time (0 day), which was set to 1. Each value represented the means of three biological replicates from three different trees with the standard error (SE) indicated by vertical bars.

To determine the possible roles of *LcAUX/IAA1*, *LcGH3.1*, *LcSAUR1*, *LcARF1* and *LcARF2* in relation to fruitlet abscission, quantitative real-time RT-PCR (qRT-PCR) analysis was performed using tissue samples from the abscission zones (AZ) and fruitlets. As shown in Figure 7, all the genes showed different expression patterns in the AZ and fruitlet, suggestive of the different roles of those genes [41]. In general, transcript levels of *LcAUX/IAA1*, *LcGH3.1* and *LcSAUR1* mRNAs were increased after the treatment of girdling plus defoliation in both AZ and fruitlet, whereas those of *LcARF1* and *LcARF2* were decreased during the same period. Although *LcAUX/IAA1*, *LcGH3.1* and *LcSAUR1* transcripts were induced by the treatment, their accumulation patterns were quite different. The level of *LcAUX/IAA1* mRNA in the AZ was increased to reach its maximum at 1 day after treatment, and markedly declined to the lowest point at 2 days, with an obvious increase observed thereafter. In contrast, the *LcAUX/IAA1* was increased gradually and peaked at 4 days in the control. In fruitlet *LcAUX/IAA1* transcript level was decreased in both the control and treated fruitlet. For the *LcGH3.1*, transcripts accumulated to a higher level in the AZ and fruitlet in response to the girdling plus defoliation application. The transcripts of *LcGH3.1* in the AZ after the treatment accumulated slowly and peaked at 4 days with approximately 2.6-fold over untreated controls whereas, in the fruitlet after the treatment, the increase of transcripts occurred at 1 days and reached the maximum level (>15-fold) at 2 days, then declined steeply afterward. In the case of *LcSAUR1*, its expression increased in the AZ under the treatment of girdling plus defoliation. The relative expression level of *LcSAUR1* also increased in the fruitlet, but this increase was much lower than that occurring in the AZ. In addition, *LcARF1* transcripts were inhibited in both the AZ and the fruitlet by girdling plus defoliation. Without the treatment, *LcARF1* transcripts decreased at 1 day and increased afterward. But when girdling plus defoliation were applied, *LcARF1* mRNAs decreased in abundance and remained constant and low at 2 to 4 days post-treatment, implying that *LcARF1* was negatively regulated by girdling plus defoliation treatment. *LcARF2*, in contrast, were nearly unaffected by the treatment and equally expressed in both the AZ and the fruitlet tissues, with a decrease at 1 day followed by a increase at 2 days and a subsequent decrease again at 4 days (Figure 7).

## 3. Discussion

Like many fruit trees species, litchi trees blossom with high profusion and thereafter exhibit massive fruitlet abscission, which leads to a low yield problem, thus limiting litchi industry development [42]. This process is generally under hormonal and metabolic regulation [43]. There is circumstantial evidence that developing fruitlets compete for photoassimilates, and the fruit abscission multiwaves are in parallel with the upsurges of abscissic acid (ABA), while IAA seems to be antagonistic [26]. Although the nature of the hormonal signals that link carbohydrate shortage and abscission are still unknown, it is commonly believed that the endogenous balance of ethylene and auxin in the AZ affects organ abscission. Application of exogenous ethylene accelerates abscission, whereas auxin treatment decreases cell sensitivity to ethylene and delays or inhibits the process [44]. Auxin exerts its effects by regulating the expression of numerous genes, including early auxin responsive genes and *ARFs*. In this paper, the isolation of *LcAUX/IAA1*, *LcGH3.1*, *LcSAUR1* and *LcARFs* from litchi fruit was firstly reported. *LcAUX/IAA1*, *LcGH3.1*, *LcSAUR1*, *LcARF1* and *LcARF2* were predicted to encode proteins of 203, 613, 142, 792 and 832 amino acids, with calculated molecular weights of 22.67, 69.20, 11.40, 88.20 and 93.16 kDa, respectively. The genes isolated have shown the highest homology to the corresponding genes: LcAUX/IAA: 70% amino acid identity with RcIAA1 (XP_002517529.1 from castor bean); LcGH3.1: 87% amino acid identity with RcGH3.6 (XP_002533739.1 from castor bean); LcSAUR1: 49% amino acid identify with PtSAUR (XP_002305471.1 from *Populus trichocarpa*); LcARF1: 79% amino acid identity with VvARF4 (XP_002285019.2 from grape); LcARF2: 78% amino acid identity with VvARF8 (XP_002266678.2 from grape). LcAUX/IAA1, like its homologs from other plant species, contains all the four conserved domains (I, II, III and IV). It is generally accepted that domain I has a repressor function [15], and domain II contributes to the rapid degradation of the Aux/IAA proteins, while domains III and IV are responsible for homo- and heterodimerization among the various members of the Aux/IAA and auxin response factor (ARF) proteins [45]. LcAUX/IAA1 may be nuclear-localized proteins due to the existence of two types of NLS (Figure 1). The protein encoded by *LcGH3.1* gene conserves three motifs essential for conjugating amino acid to excessive IAA (Figure 2) [11]. Apart from IAA, some *GH3* genes from *Arabidopsis* are also known to adenylate other plant hormones such as jasmonic acid and salicylic acid [35]. The deduced ORF of *LcSAUR1* encodes small proteins with molecular mass of 11.40 kDa, similar with those of OsSAURs, which ranged from 10 kDa for OsSAUR1 to 27 kDa for OsSAUR21 (Figure 3) [46]. The multiple sequence alignments of the full-length protein sequences showed that LcSAUR1 contains a conserved, SAUR-specific domain (SSD) of approximately 60 residues in the central region. In addition, alignment of the LcARFs amino acid sequences with that of other reported ARFs revealed that the predicted proteins shared typical features including an *N*-terminal DNA-binding domain and two *C*-terminal domains, “Box III” and “Box IV” (Figure 4), which contribute to protein dimerization [14,15].

It has been proposed that carbohydrates and hormones participate in a complex signal transduction system [33,47]. Previous work demonstrated that defoliation and girdling management could reduce the amount of resources available for current reproduction in individual branches, resulting in decrease of fruit production [32]. In addition, defoliation or shading reduces photosynthesis but also enhanced levels of abscisic acid (ABA) and 1-aminocyclo-propane-1-carboxylic acid (ACC) that positively correlated with abscission [26,47]. In the present work, we demonstrated that treatment of girdling plus defoliation reduced endogenous IAA concentration concomitant with increasing fruitlet abscission (Figures 5 and 6) and that, in litchi fruit, auxin has a key role in fruit retention. None of the litchi early auxin responsive genes or *ARFs* was AZ specific and they were also detected in the whole fruitlet. The expression patterns of auxin-related genes differed during litchi fruit AZ activation. The expression of *LcAUX/IAA1*, *LcGH3.1* and *LcSAUR1* increased in AZ and fruitlet after treatment with girdling plus defoliation which significantly induced litchi fruitlet shedding (Figure 7). By comparing the expression levels of the *LcAUX/IAA1*, *LcGH3.1* and *LcSAUR1* in the different tissues and IAA content, it is likely that the *LcAUX/IAA1* and *LcSAUR1* may play a more important role in abscission than the *LcGH3.1*, because the *LcAUX/IAA1* and the *LcSAUR1* were expressed mostly in AZ. However, these results differed from the finding of tomato flower pedicel abscission. During the first 4 h after flower excision, most *SlIAA* genes in the control reached their peak expression level at 1 h but then decreased sharply, which corresponded well to the changes of endogenous IAA level [21]. Moreover, tomato *GH3* increased slightly after 8 h and maintained a low expression level during abscission, implying that it may be an effective negative regulator in IAA-induced delay in abscission, while small auxin-up RNA was suggested to be a marker of IAA level throughout the abscission process [21]. Additionally, application of 2,4-D, which delayed floret abscission, induced a higher expression of Aux/IAA genes in the floret AZ [21]. Our findings, together with previous reports, suggest that functional divergence underlying abscission processes may be present between litchi and tomato. Thus, further studies are needed to fully unravel the function of *LcAUX/IAA1* and *LcSAUR1* genes in regulating fruit abscission.

*LcARF1* and *LcARF2* exhibited similar expression patterns both in AZ and fruitlet tissues (Figure 7). In particular, *LcARF1* was negatively correlated with fruitlet abscission in that its transcript decreased in AZ and fruitlet under the treatment of girdling and defoliation, which was contrary to the work of [22] showing that an increase of *ARF2* mRNA in detached leaves in *Arabidopsis thaliana* following dark treatment, with a time course similar to that of SAG12 mRNA. Interestingly, the expression pattern in AZ during abscission of LcARF2 was decreased at 1 days and then increased at 2 days and finally decreased again at 4 days, which is opposite of that of LcAUX/IAA1, suggesting that a cascade of AUX/IAA-ARF might also exist in litchi fruit. The developmental specificity of auxin response is determined by the interacting pairs of ARFs and Aux/IAAs [48]. Among the auxin-signaling, AUX/IAAs are proposed to bind to ARFs and inhibit transcription of *ARF* target genes, while a degradation of AUX/IAAs in response to auxin treatment would lead to increase ARF activity [49–51]. It should be needed to examine whether LcAUX/IAA1 is able to interact with LcARF2 in the future.

## 4. Experimental Sections

### 4.1. Plant Materials and Treatment

Three randomly selected 10-year-old litchi trees (*Litchi chinensis* Sonn. cv. kulin) growing in an orchard in South China Agricultural University were chosen for this study. Twenty fruit-bearing shoots located in different directions of each tree were tagged. Ten of them were treated with girdling (removing bark by about 0.5 cm in width) followed by defoliation (removing all leaves above the girdling site) at 25 days after anthesis, while the remaining untreated shoots were used as controls. Sampling was conducted at 0, 1, 2 and 4 days after treatment. Fruitlets and fruit abscission zone were collected immediately. Fruit abscission zones were excised by cutting ~1 mm at each side of the abscission fracture plane. After separation, all tissues were quickly frozen in liquid nitrogen and stored at −80 °C for future analysis. Each tree was treated as a biological replicate.

### 4.2. RNA Isolation and cDNA Cloning

Total RNA was extracted from the frozen fruitlets (~10 g) and abscission zone (~100 mg) sample according to the method of [52] and [53], respectively. RNA quality was determined by denaturing agarose gel [1.2% (w/v) agarose, 0.5 × 3-(N-morpholino)propanesulfonic acid (MOPS)] and spectrophotometer. Following the RNA extraction, potentially contaminating DNA was removed by the treatment with DNase I digestion using the RNase-free kit (Promega, Madison, WI, USA). The DNA-free total RNA was then used as template for reverse transcription-PCR (RT-PCR). The first-strand cDNA was subjected to PCR amplification. To isolate *AUX/IAA*, *GH3*, *SAUR* and *ARF* orthologs from litchi fruit, degenerate primers were designed based on available sequence information in Genbank (Table 1). PCR reactions were carried out using 0.3 μL LA Taq DNA Polymerase (Takara, Dalian Division, China) under different annealing temperatures, extension times and cycles according to the melting point of primer pairs, and the amplified fragments were cloned into a pGEM-T Easy vector (Promega, USA), sequenced, and compared with Genbank sequences using the Blast program. Extension of partial cDNA segments was carried out using the 3′- and 5′-Rapid Amplification of cDNA ends (RACE) kit (Takara, Dalian Division, China) according to the manufacturer’s instructions. The products obtained from 3′- and 5′-RACE were cloned and determined, as described above. Full-Length cDNA sequences of *LcAUX/IAA1*, *LcGH3.1*, *LcSAUR1*, *LcARF1* and *LcARF2* were assembled through sequential ligation and cloning, and verified by DNA sequencing. Deduced molecular weights and the theoretical isoelectric points of proteins were computed using the ProtParam tool. Multiple alignments were performed using ClustalX and GeneDoc program.

### 4.3. Abscission Rate Evaluation

To determine fruit abscission rate, fruit number on the tagged shoots were counted at each sampling date. Relative fruitlet abscission rate was calculated by subtracting the number of remaining fruitlets from the last recorded number, dividing by the last number and multiplying by 100. Cumulative fruit abscission was calculated by subtracting the number of remaining fruitlets from the initial number, dividing by the initial number, and multiplying by 100. Final results were expressed as percentage. Means and standard errors were calculated on a per-tree basis.

### 4.4. Measurement of Endogenous IAA Content

The IAA content was measured using the 3-indoleacetic acid (IAA) ELISA kit as previously described by [54] and [55], which has been widely used to measure IAA contents in various tissues. Tissues of fruitlet (300 mg) were sampled with a mortar and pestle under liquid nitrogen. IAA was extracted by 80% methanol and centrifuged at 5000 g for 10 min at 4 °C. The supernatant was collected and immediately passed through a pre-equilibrated C18 Sep-Pak cartridge (Millipore). The efflux was collected, and was dried in N_2_ gas. The residue was dissolved in 100% methanol for methylation with freshly synthesized ethereal diazomethane. The solution was then dried under N_2_ gas and redissolved in 300 μL of phosphate buffered saline (PBS) for IAA ELISA using a monoclonal antibody of high specificity for IAA methyl ester. In a standard procedure, both a recovery assay (using an internal control) and a dilution assay (making a series of sample extract dilutions) were performed, the first to ensure good sample recovery and the second to guarantee the absence of nonspecific inhibitors in the extracts. IAA was determined three times on the same extract, and samples were assayed in three biological replicates from three different trees.

### 4.5. Quantitative Real-Time PCR Analysis

RNA isolation and first-strand cDNA synthesis were performed as described previously. The synthesized cDNA was diluted 1:40 with water, and 2 μL of the diluted cDNA was used as a template for quantitative real-time PCR analysis (qPCR). PCRs were performed in a total volume of 20 μL, containing 1 μL of each primer (10 μM; final concentration 500 nM) and 10 μL of SYBR^®^ Green PCR Supermix (Bio-Rad Laboratories) on a Bio-Rad CFX96 Real-Time PCR System according to the manufacturer’s instructions. The qPCR program included an initial denaturation step at 94 °C for 5 min, followed by 40 cycles of 10 s at 94 °C, 30 s at 60 °C, and 30 s at 72 °C. No-Template controls for each primer pair were included in each run. The oligonucleotide primers for qPCR analysis were designed on the basis of the 3′-untranslated region using Primer 5.0 software. Each assay using the gene-specific primers amplified a single product of the correct size with high PCR efficiency (90–110%). All qPCRs were normalized using the cycle threshold (*C*t) value corresponding to the reference gene. The relative expression levels of the target gene were calculated using the formula 2^−ΔΔ^*C*t [56]. Results are representative of three biological replicates. *LcACTIN* was selected as a reference gene according to our previous study on the selection of reliable reference genes for expression study by RT-qPCR in litchi fruit [57]. The gene characteristics and their primer sequences are listed in Table 2.

## 5. Conclusions

In conclusion, one AUX/IAA (*LcAUX/IAA1*), one GH3 (*LcGH3.1*), one SAUR (*LcSAUR1*) and two ARF (*LcARF1* and *LcARF2*) genes were isolated and characterized from litchi fruit for the first time. Expression studies of these genes revealed different expression patterns in response to girdling plus defoliation application which induced fruitlet abscission of litchi and reduced endogenous IAA content. By comparing these genes’ expression levels, IAA content and fruitlet abscission, it was postulated that *LcAUX/IAA1*, *LcSAUR1* and *LcARF1* may play a more important role in the abscission of litchi fruitlet. These results provide novel molecular insight into carbohydrate stress-induced fruitlet abscission and the auxin signal transduction pathway involved in litchi fruitlet abscission.

## Figures and Tables

**Table 1:** Primers used for cloning *LcAUX/IAA1*, *LcGH3.1*, *LcSAUR1*, *LcARF1* and *LcARF2*.

Name	Sequences (5′-3′)
*AUX/IAA-For*	GGTGGTGCGCTGGCGNCCNRT
*AUX/IAA-Rev*	CGATCGCCTCGGACCGYTTNATDAT
*GH3-For*	CTACGACACCTACCARCAGWTSTAYWSNCA
*GH3-Rev*	CAGCTCCCAGAAGATCAYGTRRTSNCCNGG
*SAUR-5RACE1*	AGGATGTACTCGAAGGTTGCCWYNTCRCANGG
*SAUR-5RACE2*	CCTCCTGCTCGAAGCCGWAYTCYTCNKC
*ARF-For*	TGGAGGTTCAGGCACATCTTCMGNGGNCARCC
*ARF-Rev*	AGCAGAACTCCTCCCATGGRTCRTCNCC
*LcAUX/IAA1-3RACE1*	GTAAAGGTGGCTGTGGATGG
*LcAUX/IAA1-3RACE2*	TGAGGAGATGTTTTCGTGCT
*LcAUX/IAA1-5RACE1*	GTCGCCATCATTGTCCTCATAAG
*LcAUX/IAA1-5RACE2*	ACATCTCCTCAAGAGCAGTCAAG
*LcGH3.1-3RACE1*	CGACCCTACGACCCTTACAA
*LcGH3.1-3RACE2*	CCTACAAGTCCTCCGTCTCG
*LcGH3.1-5RACE1*	CACAGAAGGGTCAGTGATTTGGG
*LcGH3.1-5RACE2*	CCAGAGGCAAAAATAGCACCAAC
*LcSAUR1-3RACE1*	GCAGGAGGTTCATCGTTCG
*LcSAUR1-3RACE2*	GCACCTGAACCACCCTCTTT
*LcARF1-3RACE1*	CGAACAAGGAAGTGGATGCT
*LcARF1-3RACE2*	GCAACCTCTGAATCCGCAAG
*LcARF1-5RACE1*	TTGCCCAACTCTGCTGGAAGGTA
*LcARF1-5RACE2*	GAGCATCCACTTCCTTGTTCGTT
*LcARF2-3RACE1*	ACCCAACTACCCCAACCTTC
*LcARF2-3RACE2*	ACGGATGAGGTCTACGCACA
*LcARF2-5RACE1*	TGTGCGTAGACCTCATCCGTTTC
*LcARF2-5RACE2*	CTTGGAAGGAAGGTTGGGGTAGT

**Table 2:** Primers used for real-time PCR.

Name	Forward primer(5′-3′)	Reverse primer(5′-3′)
*LcAUX/IAA1*	TGAAGCAACAATAAGTGGAAAAGG	TTCGCGTGAAACAAGATGTTGTA
*LcGH3.1*	GCCACACTGCTGATAGAGAGACT	TCCCCTCCTGATGTCCCAGAACT
*LcSAUR1*	AGTGCAACAAGATTCGTCACATTG	GCGCTGCCGATGACCCTCTAGAC
*LcARF1*	CAAAGAAGAAGTGGAGAAGATGAC	ATGTAACTAGGTTCAGGTTTTCAC
*LcARF2*	AGGGGTTGAATCCTTAAATCCAAG	ATTTTCGTGGGATTATGTTATGTC
*LcACTIN*	ACCGTATGAGCAAGGAAATCACTG	TCGTCGTACTCACCCTTTGAAATC
